# Pharmacokinetic effects of endoscopic gastric decontamination for multidrug gastric pharmacobezoars

**DOI:** 10.1016/j.toxrep.2024.101683

**Published:** 2024-06-21

**Authors:** M. Marano, BM Goffredo, S. Faraci, F. Torroni, Sharada H. Gowda, S. Perdichizzi, M. Di Nardo

**Affiliations:** a Pediatric Poison Control Center, Bambino Gesù Children’s Hospital, IRCCS, Rome, Italy; b Pediatric Intensive Care Unit, Bambino Gesù Children’s Hospital, IRCCS, Rome, Italy; c Division of Metabolic Biochemistry, Children’s Hospital Bambino Bambino Gesù, IRCCS, Rome, Italy; d Digestive Endoscopy and Surgery Unit, Bambino Gesù Children’s Hospital, IRCCS, Rome, Italy; e Department of Pediatrics, Baylor College of Medicine, Texas Children’s Hospital, USA

**Keywords:** Pediatric poisoning, Endoscopic decontamination, Adolescent self-harm, Gastric decontamination, Pharmacobezoar

## Abstract

**Introduction:**

Intentional multiple drugs overdose is an often-encountered method of self-harm in adolescence. Treatments include supportive therapy, antidotes (when available) and decontamination techniques with the aim of reducing drugs absorption by the gastrointestinal system to minimize toxicity. Nevertheless, the decontamination techniques currently used, such as gastric lavage (GL), activated charcoal or whole-bowel irrigation, have a questionable effectiveness. Endoscopic gastric decontamination (EGD) treatment for massive ingestion of drugs or formation of pharmacobezoars is currently described only in anecdotal cases. Here we describe the management of an intentional drug overdose in an adolescent patient treated with EGD and the effects of this therapy on drugs pharmacokinetics.

**Case report:**

A 15-year-old boy was admitted in an unconscious state (Glasgow Coma Scale: 7–8) to the pediatric intensive care unit after assuming an unspecified amount of quetiapine, aspirin, bisoprolol, fluoxetine, furosemide, alprazolam, and pregabalin pills. Rapid sequence intubation was immediately performed and then the patient was treated with symptomatic therapy and GL with minimal removal of gastric material. Accounting for the type of drugs, the time elapsed from oral assumption and the unknown quantity assumed, EGD was attempted with aim of removing potential aggregate of the drugs. Serial blood samples were taken before and after EGD to measure the plasma level of the drugs. A pharmacobezoar was found and was immediately removed with EGD. The results of the drug monitoring showed that quetiapine exceeded the toxic level reported in literature indicating that it may have been the drug assumed in higher quantity by our patient. PICU stay was uneventful, and the patient was transferred to the psychiatric ward after extubation.

**Discussion:**

Our case shows how GL is not effective in mitigating multidrug absorption especially drugs potentially inducing pharmacobezoars. Furthermore, based on our plasma drug monitoring, we believe that early EGD should be considered in all cases of massive pill intake, prolonged release drugs that can form pharmacobezoars or in cases where a life-threatening dose cannot be excluded.

## Highlights

• The current decontamination techniques (gastric lavage, activated charcoal and whole-bowel irrigation) do not grant the complete removal of the ingested xenobiotics and can be associated to adverse events.
• Endoscopic gastric decontamination should be considered in case of prolonged released drugs potentially developing pharmacobezoars.
• Endoscopic gastric decontamination should be considered in case of massive pills ingestion.
• Endoscopic gastric decontamination should be in doubtful cases where life-threatening doses cannot be excluded.

## 1 Introduction

Intention to harm and intentional self-poisoning are the most common causes of intoxication in adolescents with multidrug ingestions representing the most common modality. When brought to the emergency room early, most patients recover with adequate clinical management. Gastrointestinal decontamination techniques (e.g. gastric lavage (GL), activated charcoal administration or whole-bowel irrigation) are commonly used to reduce the absorption of xenobiotics, limiting the onset of symptoms. Nevertheless, their effectiveness is still debated. Currently, GL is utilized as an initial therapeutic technique to remove xenobiotics within the first hour of ingestion. The main limitation of GL is the incomplete removal of xenobiotics, the inability of the patient to tolerate the administration of large volume of fluid and, the size of the Levine probe. Inherent complications of this procedure include lesions in the esophageal and gastric mucosa, bleeding etc. Thus, for all these reasons it must be carried out by experts. Therefore, until methodologically valid clinical studies are published, this technique should not be performed routinely, but evaluated on a case-by-case basis [1].

Activated charcoal is an insoluble powder created by the pyrolysis of various organic materials (peat, wood, coconut) oxidized at temperatures between 500 and 900°C. It has a great absorbent capacity. Easy to administer (less so in younger children), in a single or multiple doses depending on the presence of the drug enterohepatic circulation. Nevertheless, it is not effective in case of drugs containing lithium, iron or in the presence of pharmacobezoars [2].

Whole bowel irrigation has the function of "freeing" the intestine and reducing the systemic absorption of the xenobiotic, but the evidence supporting this technique as a beneficial treatment for poisoned patients is weak, and thus this procedure is not be performed routine [3].

Endoscopic gastric decontamination (EGD) is an advanced approach recently described in children to provide gastric decontamination in case of massive pills ingestions or potential bezoar [4], [5]. This technique is underused in pediatric multidrug overdose ingestions due to lack of data on its beneficial pharmacokinetic effects. Here, we describe the successful utilization of EGD in a pediatric patient with multidrug ingestion with emphasis on the pharmacokinetic effects of the ingested drugs.

## 2 Case report

**Fig. 1:**
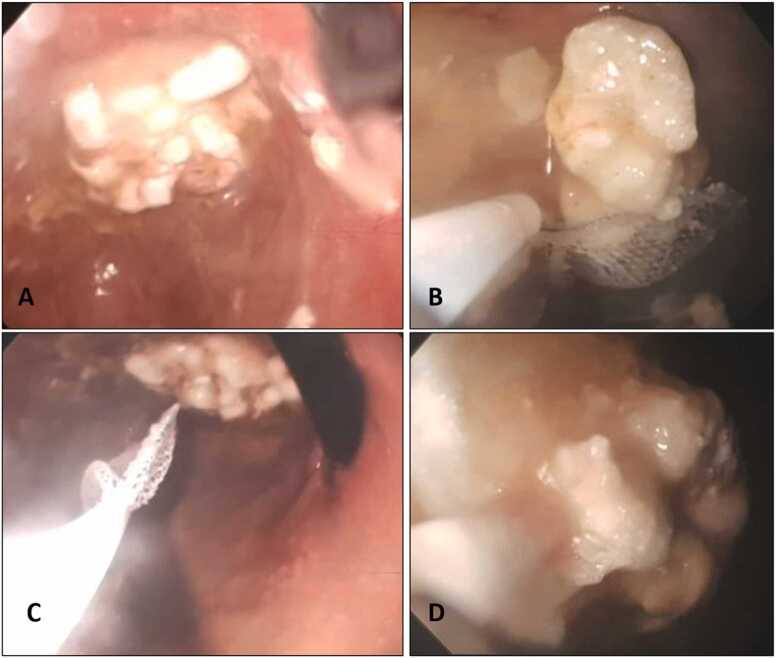
Pharmacobezoar diagnosis (A) and pharmacobezoar removal with a wire-basket during gastric endoscopy (B-D).

A 15-year-old adolescent girl (48 kg body weight) was admitted to the Pediatric Intensive Care Unit (PICU) three hours after an unwitnessed ingestion of an unknown amount of quetiapine (slow-release formulation), aspirin, bisoprolol, fluoxetine, furosemide, alprazolam, pregabalin. Upon arrival, she was unresponsive (Glasgow Coma Scale: 7–8) with sluggishly reactive pupils and bradypnea, thus, intubation was immediately performed to secure the airways. Pulmonary examination and chest X-ray were normal. The electrocardiogram showed a sinus bradycardia (65 beats/min) with a QTc duration of 460 msec. Blood pressure was 85/40 mmHg and was effectively treated with fluid resuscitation and noradrenaline (0.03 mcg/kg/min). Brain computed tomography scan was negative. Laboratory findings including blood gas analysis and lactate levels were within normal limits. Considering the potential delayed gastric emptying of quetiapine, we attempted GL with minimal success in removing the ingested materials. In consideration of the type of drugs, the time elapsed from assumption and the unknown quantity assumed by the patient, EGD was performed as soon as the endoscopy team was available in hospital (two hours after PICU admission) and the parents gave their consent. A pharmacobezoar was found and totally removed with a wire basket (Fig. 1 A-D).

**Fig. 2:**
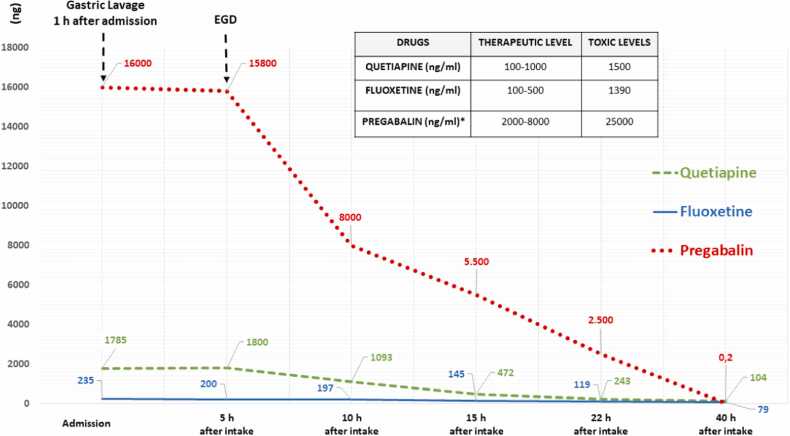
Plasma levels of quetiapine, fluoxetine and pregabalin during pediatric intensive care unit stay (EGD: endoscopic gastric decontamination, h: hour). *Micrograms have been converted to nanograms in the graph.

Plasma samples for drugs monitoring were collected before and after EGD at the 4th, 5th, 10 ^th^, 15 ^th^, 22 ^th^, and 40 ^th^ hour from drug ingestion. Quetiapine, bisoprolol, pregabalin and fluoxetine were the only drugs identified in our laboratory (Fig. 2). Drug levels were measured with liquid chromatography and mass spectrometry using a UHPLC Agilent 1290 Infinity II 6470 (Agilent Technologies, Santa Clara, CA 95051, United States) equipped with an ESI-JET-STREAM source operating in the positive ion (ESI+) mode. The samples were analyzed using a validated LC-MS/MS kit (MassTox® TDM Anti-arrhythmic drugs, Neuroleptics, Antidepressants and Antiepileptic Drugs) provided by Chromsystems (Chromsystems Instruments & Chemicals GmbH, 82166 Gräfelfing/Munich, Germany). This kit was validated by the European Medicines Agency.

The PICU stay after EGD was uneventful, and the patient was extubated after 48 h from the ingestion. The following day was transferred to the neuropsychiatric ward.

## 3 Discussion

The therapeutic concentrations of quetiapine range between 100 and 1000 ng/ml, while toxic levels are described over 1500 ng/ml [6]. The therapeutic concentrations of fluoxetine range between 50–480 ng/ml, while minimal toxic effects are described up to 1390 ng/ml (fluoxetine plus norfluoxetine) [7]. The therapeutic concentrations of pregabalin range between 2.8 and 8.2 mcg/ml, while toxic levels are described over 25 mcg/ml [8] The therapeutic concentration of bisoprolol, instead, range between 0.01 and 0.06 mg/L, while toxic levels are described over 7.54 mg/L [9].

Pharmacokinetic data from literature suggest that quetiapine reaches its peak plasma concentration 60–90 minutes after ingestion and has an elimination half-life of almost six hours, however, potential side effects may persist even after two days from the ingestion due to the slow elimination from the stomach [10]. Fluoxetine reaches its peak plasma concentration 4–6 hours after ingestion and has an elimination half-life of approximately four days. Pregabalin reaches its peak plasma concentration 1–3 hours after ingestion and has an elimination half-life between six and eight hours. Bisoprolol is rapidly adsorbed after ingestion, and it reaches peak plasma concentrations after two hours. Its half-life is of 10–12 hours. Nevertheless, pharmacokinetics characteristics of drugs assumed in high quantities or, potentially developing bezoars, may be different from the ones assumed for therapeutic purposes [4].

In our patient, the plasma levels of quetiapine, fluoxetine and pregabalin, evaluated after the gastric lavage were high, but only quetiapine reached toxic levels (1800 ng/ml). The plasma levels of bisoprolol, instead, were below normal limits, therefore were not reported in Fig. 2. Nevertheless, several factors may have contributed to the development of the pharmacobezoar: a) the type of drug assumed (e.g. quetiapine), b) the large quantity of pills assumed and, c) the anticholinergic effect of the drugs (e.g. quetiapine) inducing impaired gastric mobility and delayed gastric emptying. Currently, there are no data on the pharmacokinetics effects of EGD due to the difficulty in obtaining levels of medications used for intentional overdose. In our patient, we were able to show that GL was not effective in removing the drugs assumed as compared with EGD and this would have led to further absorption of the drugs taken with the potential worsening of the symptoms described above.

Drug monitoring after EGD showed that only quetiapine reached toxic plasma levels and perhaps it was the most prevalent drug assumed by the patient. The other drugs (pregabalin and fluoxetine) progressively decreased their plasma levels when the bezoar was removed with EGD (Fig. 2).

Based on these pharmacologic data, we believe that early EGD should be considered in all cases of massive pills ingestion, prolonged released drugs, in case of drugs potentially developing pharmacobezoars or, in doubtful cases where life-threatening doses cannot be excluded.

## Informed consent

Informed consent for publication was obtained from the parents of the patient.

## CRediT authorship contribution statement

**Matteo Di Nardo:** Data curation. **Marco Marano:** Conceptualization. **Bianca Maria Goffredo:** Methodology. **Simona Faraci:** Investigation. **Filippo Torroni:** Investigation. **Sharada H. Gowda:** Formal analysis. **Salvatore Perdichizzi:** Conceptualization.

## Declaration of Competing Interest

The authors declare that they have no known competing financial interests or personal relationships that could have appeared to influence the work reported in this paper.

## Data Availability

Data will be made available on request.
